# The crux of NPO paradox revealed by increased pneumonia incidence in post-stroke dysphagia patients with dietary restrictions

**DOI:** 10.1038/s41598-025-22020-3

**Published:** 2025-10-03

**Authors:** Manuela Ihrke, Andreas Meisel, Alexander Nelde, Konrad Neumann, Dirk Mürbe, Linda J. Voß, Philipp P. Caffier

**Affiliations:** 1 Department of Audiology and Phoniatrics, Charité – Universitätsmedizin Berlin, Campus Benjamin Franklin, Corporate Member of Freie Universität Berlin and Humboldt-Universität Zu Berlin, Hindenburgdamm 30, 12203 Berlin, Germany; 2 Center for Stroke Research Berlin, Charité – Universitätsmedizin Berlin, Charitéplatz 1, 10117 Berlin, Germany; 3 Department of Neurology with Experimental Neurology, Charité – Universitätsmedizin Berlin, Bonhoefferweg 3, 10117 Berlin, Germany; 4 Berlin Institute of Health at Charité – Universitätsmedizin Berlin, Anna-Louisa-Karsch-Str. 2, 10178 Berlin, Germany; 5 Institute of Biometry and Clinical Epidemiology, Charité – Universitätsmedizin Berlin, Campus Charité Mitte, Charitéplatz 1, 10117 Berlin, Germany

**Keywords:** Stroke-associated pneumonia, Post-stroke dysphagia, Nil per os, Texture modified food, Stroke unit, Dysphagia management, Diseases, Neurological disorders, Stroke, Risk factors, Nutrition, Respiratory signs and symptoms

## Abstract

Dysphagia patients are at increased risk of stroke-associated pneumonia (SAP). This bicenter study evaluated whether dietary restrictions, specifically nil per os (NPO) and texture-modified food (TMF), reduce SAP incidence in post-stroke dysphagia (PSD). Data from 160 consecutive acute PSD patients treated in two university stroke units were retrospectively analyzed. The primary outcome was SAP incidence; secondary outcomes included length of hospitalization, mortality, and nasogastric tube placement. Stroke severity, male sex, and age emerged as significant SAP risk factors. On admission, 63% of SAP patients were already on NPO, 33.3% received TMF, and only 3.7% had unrestricted oral intake. Notably, NPO patients were 2.5 times more likely to develop SAP than those without dietary restrictions (*p* = 0.008). Most SAP cases were diagnosed before any oral intake, with the majority occurring by day three. These findings align with emerging evidence highlighting the role of oral hygiene factors and immune alterations in pulmonary bacterial defense. This study provides no support for NPO or TMF as effective pneumonia prophylaxis in PSD. Instead, early intervention and comprehensive care strategies are essential to mitigate SAP risk. Appropriate dysphagia diets enhancing residual swallowing capacity could positively impact both SAP rates and dysphagia rehabilitation.

## Introduction

Stroke is a clinical syndrome characterized by acute neurological deficits resulting from vascular disorders, including infarction and hemorrhage, that affect the central nervous system^1,2^. Worldwide, around 15 million individuals experience a stroke each year, leading to approximately 5 million deaths and 5 million permanent disabilities, which places an enormous burden on those affected, their families and the community^3,4^. The incidence of post-stroke dysphagia (PSD) among acute stroke patients ranges from 29 to 81%, depending on the diagnostic criteria, timing of assessments, and type of testing procedures^5^. It is estimated that approximately 50% of all stroke patients develop a swallowing disorder within the first 72 h post-stroke^6^. PSD compromises the safety of food intake, elevating risks for aspiration pneumonia, malnutrition, dehydration, and dependence on tube feeding^5,7,8^. Affected individuals are particularly susceptible to develop stroke-associated pneumonia (SAP) due to aspiration of food or infectious saliva into the lower respiratory tract^9^. Additionally, PSD contributes to a reduced quality of life, depression, and social withdrawal^10–12^.

SAP is the most common preventable complication following stroke in the acute phase, typically occurring between days 2 and 7 post-event, with an average onset around day 4.7^13,14^. The incidence of SAP ranges from 7 to 22%, with patients experiencing dysphagia at the highest risk: PSD can increase the SAP likelihood by up to 11 times^5,6^. Several risk stratification scores and tools have been introduced for SAP prediction^15–17^. Hoffmann et al. (2012) analyzed data from the Berlin Stroke Registry to develop the A^2^DS^2^ score^18^, which incorporates the relevant factors age, atrial fibrillation, dysphagia, male sex, and stroke severity. It calculates the cumulative SAP risk, with higher scores indicating increased risk, ranging from a 0.3% SAP rate at a score of 0, to 39.4% at a score of 10. However, this study did not provide a detailed description of dysphagia symptoms. In addition to dysphagia, other significant risk factors for aspiration pneumonia in stroke patients include impaired consciousness, reduced bulbar reflexes, prolonged bed rest, and invasive medical interventions^15,19^.

SAP is consistently linked to increased mortality rates^18–20^, with dysphagic patients being most vulnerable^6^. Consequently, there is both ethical and economic incentive to rapidly identify at-risk groups and implement appropriate interventions^21,22^. Texture-modified food (TMF) and nil per os (NPO) are commonly applied interventions in PSD, although their effectiveness in preventing SAP remains controversial^23^. TMF aims to enhance swallowing safety by altering bolus properties such as viscosity, volume, and texture. Recent reviews confirm that thickened liquids can reduce penetration/ aspiration events by slowing bolus transit and improving airway protection, but they are also associated with increased post-deglutitive residues, dehydration, malnutrition, and reduced quality of life^24^. Observational studies report that TMF can facilitate safe oral intake, improve nutritional status, and prevent the need for feeding tubes in the acute phase of stroke^25,26^. However, robust evidence demonstrating a reduction in SAP incidence is lacking, as no randomized controlled trials (RCTs) have confirmed this effect.

NPO is widely used in acute stroke protocols to prevent aspiration until swallowing safety is established through formal assessment. Despite its routine use, recent studies show that NPO does not reliably reduce SAP incidence. Teuschl et al. observed that patients kept NPO until screening still developed pneumonia, suggesting that silent aspiration of saliva or refluxate remains a significant risk^27^. Furthermore, prolonged NPO is associated with dehydration, malnutrition, and delayed swallowing rehabilitation^28,29^. Taken together, both TMF and NPO are widely practiced strategies with physiological rationale but limited clinical evidence supporting their effectiveness in pneumonia prevention. The absence of high-quality RCTs leaves a significant knowledge gap.

In one of our preceding investigations evaluating TMF according to the International Dysphagia Diet Standardisation Initiative (IDDSI)^30^, it was observed that dietary modifications had a limited impact on pneumonia rates within the stroke unit, as it was mainly patients under NPO who suffered pneumonia^31^. This raises questions about the efficacy of NPO and TMF as prophylactic measures against SAP. The aim of our present investigation was therefore to explore the association between different feeding strategies and the likelihood of developing SAP in more detail. To our knowledge, this is the first bicenter study in two university tertiary care institutions comparing pneumonia rates for NPO, TMF and regular diet in consecutively admitted acute stroke patients over a 20-month period.

## Material and methods

### Study design and eligibility

This retrospective observational cohort study was conducted in accordance with the Declaration of Helsinki and on approval by the Ethics Committee of Charité—Universitätsmedizin Berlin (EA1/186/23). The design was chosen due to the feasibility of analyzing existing clinical records and the availability of comprehensive historical data from routine care to evaluate texture-modified diets based on IDDSI guidelines. The retrospective data review included acute PSD patients who were hospitalized at two university tertiary care centers. The dataset was analyzed to investigate the association between diet and the incidence of pneumonia in post-stroke patients, regardless of the specific type of TMF provided. Since only routine clinical data were examined, the Charité Institutional Review Board waived the requirement to obtain informed consent for study participation.

### Patient sample

The patients were treated in two stroke units at Charité Campus Benjamin Franklin (CBF) and Charité Campus Mitte (CCM), each with a total capacity of 20 beds. Inclusion criteria comprised confirmed intracerebral haemorrhage or cerebral infarction, inpatient stay on the stroke unit, confirmed dysphagia, as well as speech-language pathologist (SLP) consultation and initiation of swallowing therapy within the first 48 h of admission (day 0–2). The periods of stay between December 1, 2021, and September 30, 2022, as well as between November 1, 2022, and August 31, 2023 were examined. Data from October 2022 were omitted, as this month marked the implementation of updated dysphagia diet protocols. Patients diagnosed with transient ischemic attack (TIA) or COVID-19 were excluded from the study.

### Dysphagia assessment

The standard operating procedure (SOP) on stroke units stipulates that patients remain on NPO after an abnormal dysphagia screening done by the nursing staff, until they receive a clinical swallow examination (CSE)^32^. This corresponds with the in-house standard, which aligns with established publications and guidelines and is conducted by a trained SLP, experienced in swallowing therapy. After obtaining the patient history and assessing cooperation and vigilance, a thorough evaluation of the oropharyngeal structures is performed, including an assessment of oral hygiene and dental status, lower cranial nerve function, secretion and salivation management, respiratory-swallowing coordination, voluntary and reflexive cough response, voice function and quality, laryngeal elevation during swallowing, oropharyngeal sensation, and spontaneous swallowing frequency. Thereafter, swallowing tests with various consistencies are carried out, typically starting with pureed, followed by liquid and solid textures. In the event of abnormal findings, swallowing maneuvers are applied to improve the safety and efficiency of the swallowing process^5,33,34^. The SLPs from both centers are part of a single, cross-site team, ensuring a standardized approach to clinical swallowing assessments across locations. Based on these results and in consultation with the treating disciplines, a dietary recommendation is made. Depending on the disorder pattern and severity, patients receive functional dysphagia therapy or measures according to Facial-Oral Tract Therapy.

### Data collection

**Fig. 1:**
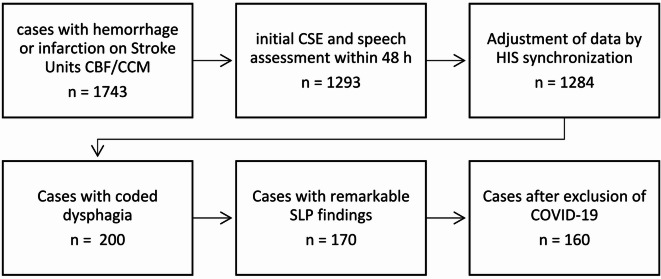
Flowchart of data collection process showing the number of patient cases found and included in the data analysis. CBF, Charité Campus Benjamin Franklin; CCM, Charité Campus Mitte; COVID-19, coronavirus disease 19; CSE, clinical swallow examination; HIS, hospital information system; SLP, speech-language pathologist.

Figure 1 illustrates the data collection procedure. Initially, anonymized stroke cases with ICD-10-GM diagnoses for intracerebral hemorrhage (I61.*) or cerebral infarction (I63.*) were extracted for the period from December 1, 2021, to August 31, 2023 (n = 1743). As planned, October 2022 was excluded from analysis due to the TMF conversion process at the university hospital. Following the application of filters to identify cases that received dysphagia assessment and therapy within the first 48 h of admission (day 0–2), the dataset was reduced by approximately 400 patients. After selecting the documented findings, the data sets were adjusted for cases with unremarkable CSE and incomplete documentation. Following the exclusion of COVID-19 cases, 160 complete data sets were finally available for further analysis. The data collection process, including the identification, screening, and selection of patient cases for inclusion in the final analysis, is detailed in Fig. 1.

Anonymized data from the Charité Data Warehouse Connect (DWC) system were investigated. The DWC platform ensures reliable and bias-free collection of clinical data and has been implemented on the neurological wards’ monitoring beds following approval from the data protection department and a completed data protection impact assessment. The DWC system facilitates the long-term storage of multi-modal (neuro) monitoring data collected in real time for all patients in the designated areas. Key physiological parameters including temperature, blood pressure, and oxygen saturation are recorded. The clinical monitoring data from the DWC platform is linked to the data lake of the Berlin Institute of Health via a data interface, allowing for subsequent visualization and analysis.

Data were transferred to SPSS using Structured Query Language (SQL) for statistical analysis. Dependent variables were examined through binary logistic regression, accounting for potential confounders such as age, biological sex, stroke severity (according to National Institutes of Health Stroke Scale (NIHSS) score), and atrial fibrillation, which were treated as independent variables. Continuous variables, including age, and inpatient length of stay (LOS), were summarized using means (M) and standard deviations (SD). Biological sex (binary), atrial fibrillation (binary), and chronic obstructive pulmonary disease (COPD) status (binary) were recorded in the DWC system. Patient age at admission (metric) was calculated using the date of birth and date of admission. The A^2^DS^2^ score (ordinal) was computed based on age, atrial fibrillation, dysphagia, NIHSS score, and biological sex.

Cases with coded dysphagia diagnosis were then de-anonymized and retrieved from the hospital information system (HIS). COVID-19 diagnoses were also extracted from HIS while pneumonia diagnoses (binary) were obtained through the HIS as determined by treating neurologists using the modified Centers for Disease Control and Prevention (CDC) criteria^35^. These require at least one systemic feature (fever > 38 °C with no other recognized cause; leukopenia < 4000 WBC/mm^3^ or leukocytosis > 12,000 WBC/mm^3^; or, in adults ≥ 70 years, altered mental status with no other cause), at least two respiratory features (e.g., new/worsening purulent sputum or respiratory secretions, new/worsening cough, dyspnea or tachypnea > 25/min, rales/crackles/bronchial breath sounds, or worsening gas exchange), and for definite SAP radiographic evidence on ≥ 2 serial chest radiographs of new or progressive and persistent infiltrate, consolidation, or cavitation.

Additionally, data on nasogastric feeding tube usage (binary), mortality (binary), and LOS in days (metric) were likewise retrieved from the aforementioned system. For binary variables (SAP, nasogastric tube usage, COPD status, COVID-19 infection, mortality), the absence of documentation was interpreted as the condition not being present. Dietary recommendations—including regular diet, TMF, and NPO—as well as any adjustments during hospitalization were extracted from the SLP therapy section of the HIS and categorized as follows: 0 = NPO, 1 = TMF, and 2 = no dietary restriction (NDR). NPO means that patients do not take any food, liquid, or medication orally, except during dysphagia therapy sessions with the SLP. In our analysis, TMF refers to a restriction of oral intake to specific consistencies or to regular food with texture modification. This includes thickened liquids, puréed and/or soft consistencies, and, if necessary, bite-sized portions (cf. IDDSI Levels 1–EC7). NDR indicates no dietary restrictions, allowing unrestricted selection from all hospital meals as well as food brought from home. Diet status was treated as a static covariate, documented on admission, at discharge, and two days before SAP. To ensure data accuracy, dietary information was randomly cross-checked by a second reviewer. Blinding during data extraction was not performed due to feasibility constraints. A list of all dependent and independent variables is summarized in Table 1.

**Table 1:** Dependent and independent variables of the analysis.

Dependent variables	Independent variables
Stroke-associated pneumonia (SAP)	A^2^DS^2^ score
Nasogastric tubes	Chronic obstructive pulmonary disease (COPD)
Inpatient length of stay (LOS)	Feeding strategy (NPO, TMF, NDR)
Mortality	Flexible endoscopic evaluation of swallowing (FEES, in SAP-cases only)

A^2^DS^2^ score, age, atrial fibrillation, dysphagia, sex, stroke severity score; NDR, no dietary restrictions; NPO, nil per os; TMF, texture modified food.

### Data analysis

All data were analyzed using IBM SPSS Statistics, Version 27.0.0.0. For statistical analysis, group differences of patient characteristics and secondary outcome parameters were assessed using the Pearson’s chi-square test for dichotomous variables (sex, COPD, nasogastric tube placement, mortality) and t-tests for metric and ordinal variables (patient age, NIHSS, LOS). Binary logistic regression models were used to assess associations of the A^2^DS^2^ score, age, sex, stroke severity, atrial fibrillation, dietary and vigilance status with the binary outcome variable (pneumonia: yes/no). Odds ratios (ORs) with two-sided 95% confidence intervals (CI) were calculated separately for all variables. This approach was chosen to avoid redundancy and multicollinearity that could arise from including both the composite score A^2^DS^2^ and its components in a single model. For the secondary outcomes (nasogastric tube placement, mortality, LOS), multiple binary or ordinal logistic regression models were applied with dietary status as the main predictor and the potential confounders A^2^DS^2^ score and COPD as covariates. Because the selection of variables was conceptually based and not data-driven, we did not formally test for multicollinearity or assess overall model fit.

For graphical visualization, bar charts and line charts were created, and FEES images were presented. A forest plot was chosen to summarize the statistical association between SAP and possible confounders using Microsoft Excel 2021, as it enables the simultaneous display of odds ratios (OR), 95% confidence intervals (CI), and p-values in a concise and comparable format. Furthermore, a Sankey flow diagram was created to illustrate the changes and proportions of different feeding strategies from admission (after initial CSE) to discharge. Sankey diagram was modified after being generated with SankeyMATIC by Steve Bogart (https://sankeymatic.com). As this study was exploratory, no adjustments were made for multiple comparisons. The significance level was set at α = 0.05.

## Results

### Cohort description

The complete dataset comprised 160 study participants, 73 male (22–95 years, median 79) and 87 female subjects (50–101 years, median 84). At hospital admission (day 0), women were on average 8 years older than men (83 ± 11 vs. 75 ± 14, mean ± SD, *p* = 0.39), which is not a significant difference. The cohort had a mean age of 80 years; 45.6% were male, 3.8% had chronic obstructive pulmonary disease (COPD), and the mean NIHSS score on admission was 11.3. More detailed, selected descriptive data of the total cohort and both subgroups of PSD patients without vs. with SAP is shown in Table 2. No statistically significant differences were found between SAP and non-SAP groups regarding age, COPD prevalence, gender distribution, or NIHSS scores, indicating baseline comparability.

**Table 2:** Descriptive data of the study cohort.

Variable	Total cohort (n = 160)	Non-SAP group (n = 106)	SAP group (n = 54)	*p*-value
Age in years; mean (SD)	80 (13)	78 (14)	82 (10)	0.114
COPD; %	3.8	4.7	1.9	0.367
Male sex; %	45.6	40.6	55.6	0.072
NIHSS score; mean (SD)	11.3 (6.9)	10.1 (6.7)	13.7 (6.7)	0.751

COPD, chronic obstructive pulmonary disease; NIHSS, National Institutes of Health Stroke Scale; SAP, stroke-associated pneumonia.

### Influencing factors for SAP

Although individual variables did not differ significantly between groups, the A^2^DS^2^ score was significantly associated with SAP risk (*p* < 0.001). The odds ratio of 1.434 (95% CI 1.18–1.75) suggests that the odds of developing SAP increase by approximately 43.4% per additional point in the A^2^DS^2^ score. Binary logistic regression identified NIHSS score (OR = 1.10 per point increase, 95% CI 1.04–1.16, *p* = 0.001), male sex (OR = 3.23, 95% CI 1.49–7.02, *p* = 0.003), and age (OR = 1.04 per year, 95% CI 1.00–1.07, *p* = 0.027) as independent risk factors for SAP development.

**Fig. 2:**
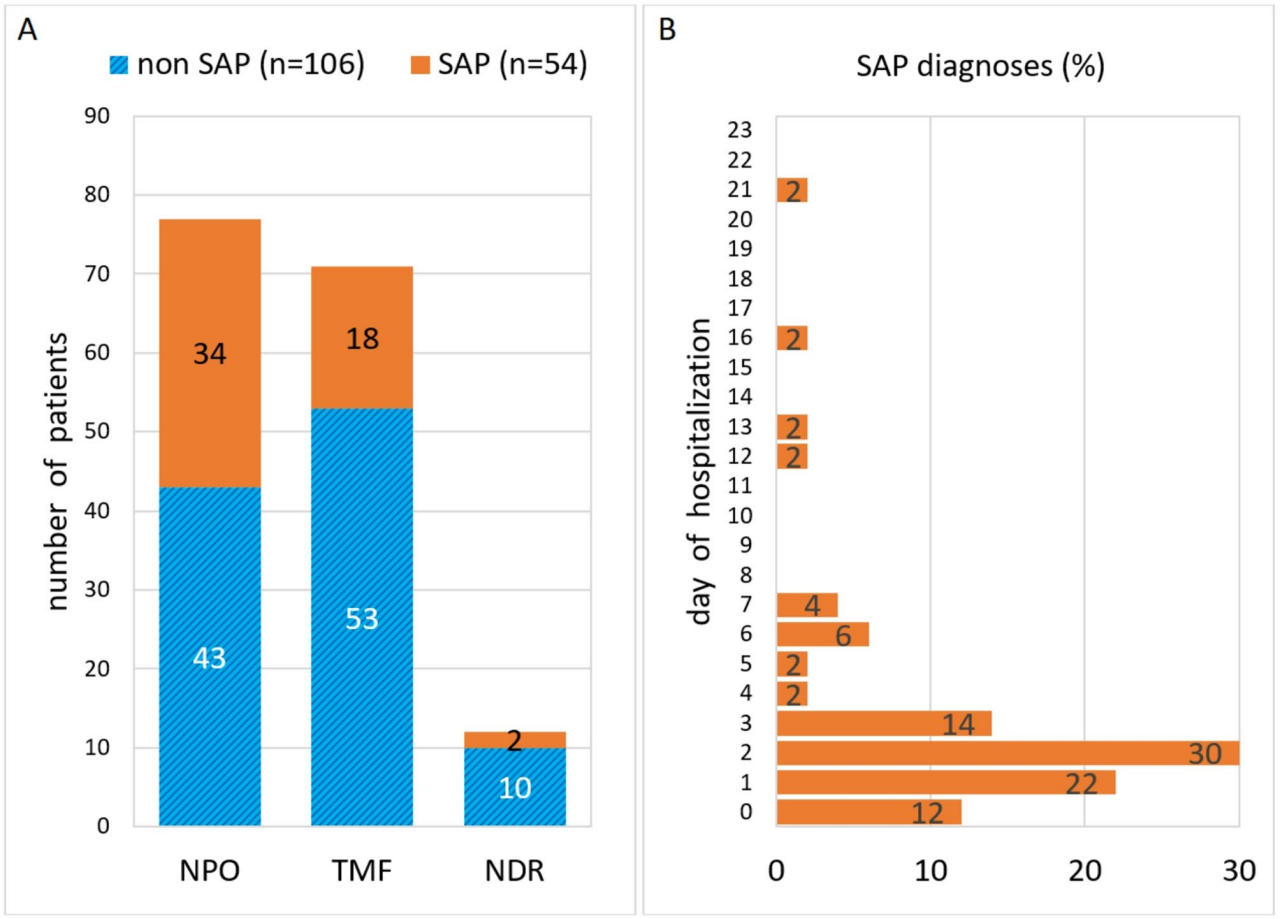
**A** (left). Distribution of stroke patients by type of diet after CSE (day 0–2 of hospitalization) categorized by SAP diagnosis. **B** (right). Distribution of SAP diagnoses by day of hospitalization in all patients with exact documentation of pneumonia diagnosis date (n = 50). CSE, clinical swallow examination; NDR, no dietary restrictions; NPO, nil per os; SAP, stroke-associated pneumonia; TMF, texture modified food.

There was also a significant association between the oral feeding route—defined as intake of food and liquids via mouth, including TMF and NDR—and the development of SAP following the CSE. Specifically, patients with NPO status exhibited a significantly higher risk of developing SAP compared to those on oral feeding routes (Pearson chi-square, *p* = 0.008). The oral feeding route was thus associated with a lower SAP incidence. The relationship between nutritional strategies and the likelihood of pneumonia in acute PSD patients is illustrated in Fig. 2A, while Fig. 2B presents the timing of SAP diagnoses during hospitalization.

PSD patients with NPO status had an approximately 2.5-fold higher odds of developing SAP compared to those on oral feeding (OR = 2.49, 95% CI 1.27–4.89, *p* = 0.008). At discharge, the differences in feeding routes became even more pronounced (*p* < 0.001), likely reflecting that the diagnosis of pneumonia influenced the dietary recommendations made by the treating SLPs. While NPO status is intended to prevent pneumonia, binary logistic regression analysis indicated that patients with oral feeding had 0.4 times lower odds of acquiring SAP compared to those on NPO (b = − 0.913, *p* = 0.008). When comparing all diets, the OR for TMF on admission (in reference to NDR) was 1.698 (95% CI 0.34–8.492; *p* = 0.519). However, the small sample size in the TMF group limits the statistical power and precision of these estimates.

In addition, more vigilance disorders and a higher mortality rate were observed in the SAP group. These differences did not reach statistical significance, potentially also due to limited statistical power. Furthermore, all non-significant ORs should be interpreted cautiously. Among the 77 NPO patients, 29 (37.7%) had initially a documented reduction in vigilance, i.e. decreased alertness or impaired ability to maintain attention. In contrast, only 4 patients (4.8%) of the cases with oral feeding routes (n = 83) exhibited reduced alertness. Since adequate vigilance is essential to ensure safe swallowing and prevent complications, initiation of oral intake required an adequate level of alertness, defined as a score of 0 (alert) or 1 (arousable by minor stimulation) on NIHSS item 1a (level of consciousness).

**Fig. 3:**
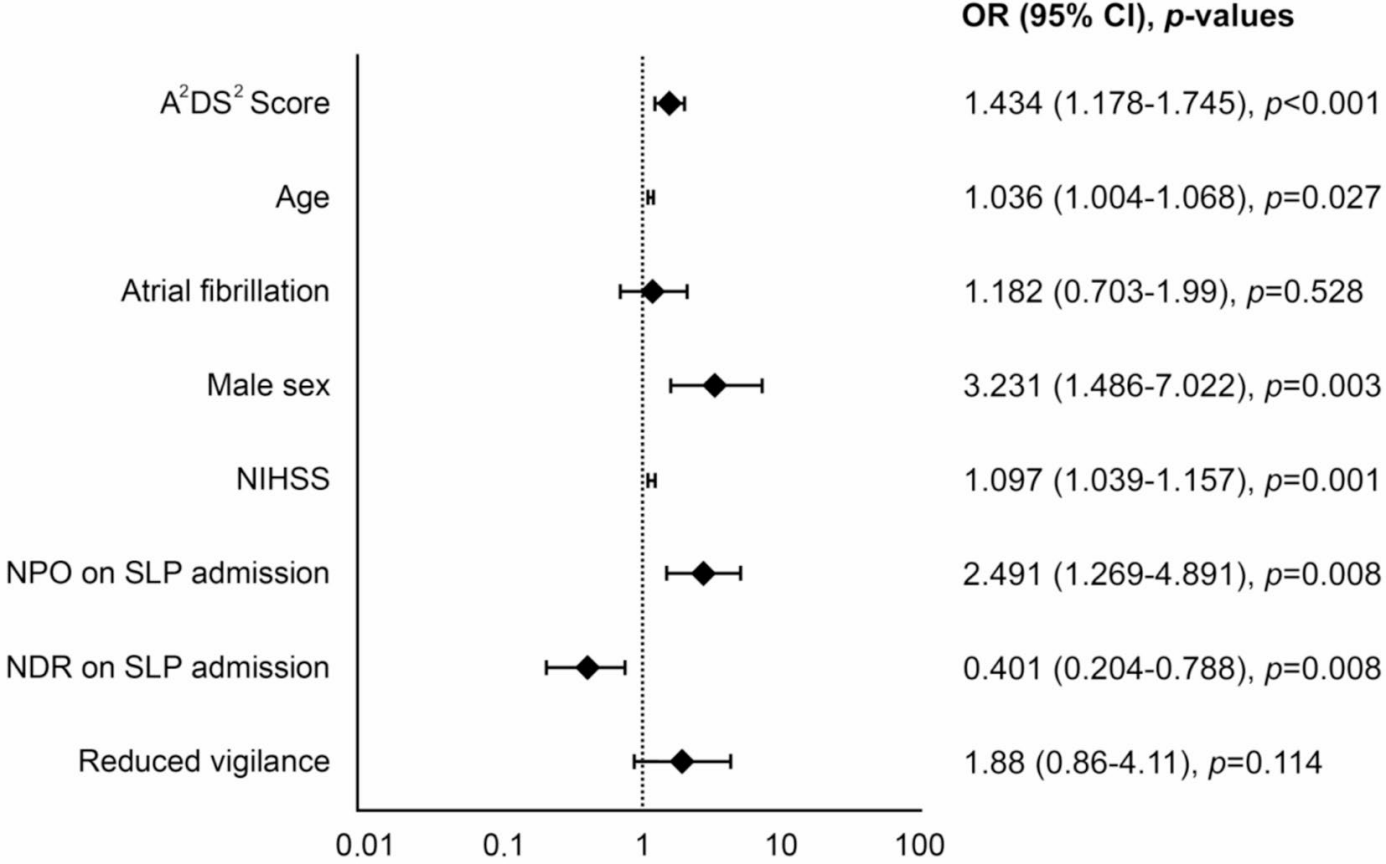
Clinical parameters predicting SAP development with odds ratios (OR), 95% confidence intervals (CI), and *p*-values. A^2^DS^2^ score, Age, Atrial fibrillation, Dysphagia, Sex, Stroke Severity score; NDR, no dietary restrictions; NIHSS, National Institutes of Health Stroke Scale; NPO, nil per os; SAP, stroke-associated pneumonia; SLP, speech-language pathologist; TMF, texture modified food.

Figure 3 presents a forest plot illustrating the clinical parameters associated with SAP development. Relevant predictors include A^2^DS^2^ score, age, male sex, NIHSS, and NPO status on SLP admission. In contrast, atrial fibrillation did not emerge as a significant factor influencing the likelihood of pneumonia. Given the observational design, these findings should be interpreted as associations rather than causal relationships. Potential mechanisms for the higher SAP risk among NPO patients may include underlying severity of neurological impairment, reduced oral clearance, or higher prevalence of vigilance disorders in this group.

Table 3 presents the dietary status after initial CSE and on discharge for the total study population, as well as for the subgroups with and without SAP. In the entire cohort, 48.1% of patients were placed on NPO status at admission, while 44.4% were on a TMF, and only 7.5% had NDR. At discharge, 30.6% PSD patients remained on oral food restrictions, while 51.9% were on TMF, and 17.5% on a regular diet. It should be noted that the discharged patients include those who had passed away. When comparing patients with and without SAP, significant differences in dietary status were observed. The proportion of NPO patients at admission was significantly higher in the SAP compared to the non-SAP group (63.0 vs. 40.6%). The difference in dietary status at discharge was even more apparent, with 78.3% of the non-SAP group on oral feeding compared to 51.9% of those with an SAP diagnosis during their hospital stay.

**Table 3:** Diet after initial CSE and on discharge. The number (n) and percentage (%) of patients are displayed for the total cohort, the non-SAP, and the SAP group.

Diet after initial CSE	Total cohort (n = 160)	Non-SAP group (n = 106)	SAP group (n = 54)
NPO	77 (48.1%)	43 (40.6%)	34 (63.0%)
Oral feeding route	83 (51.9%)	63 (59.4%)	20 (37.0%)
TMF	71 (44.4%)	53 (50.0%)	18 (33.3%)
NDR	12 (7.5%)	10 (9.4%)	2 (3.7%)

Diet on discharge	Total cohort (n = 160)	Non-SAP group (n = 106)	SAP group (n = 54)
NPO	49 (30.6%)	23 (21.7%)	26 (48.1%)
Oral feeding route	111 (69.4%)	83 (78.3%)	28 (51.9%)
TMF	83 (51.9%)	60 (56.6%)	23 (42.6%)
NDR	28 (17.5%)	23 (21.7%)	5 (9.3%)

CSE, clinical swallow examination; NDR, no dietary restrictions; NPO, nil per os; SAP, stroke-associated pneumonia; TMF, texture modified food.

**Fig. 4:**
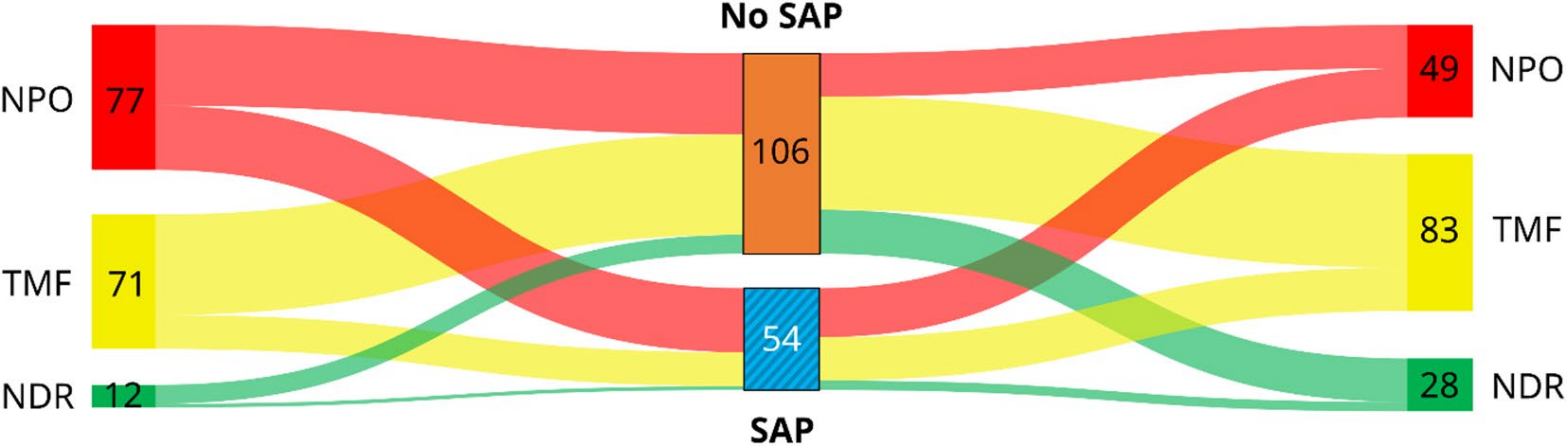
Different feeding strategies after initial CSE (left) and at discharge (right) in the total cohort, with visualization of changes over time and SAP development. CSE, clinical swallow examination; NDR, no dietary restrictions; NPO, nil per os; SAP, stroke-associated pneumonia; TMF, texture modified food.

The different courses of dietary recommendations for patients with and without SAP are shown as a flow analysis in Fig. 4. In the Sankey diagram, the width of the colored bands is proportional to the number of patients with different feeding strategies over the duration of their inpatient LOS. This indicates that among the dietary regimes, patients on NPO status showed the greatest association with SAP development.

### Analysis of SAP group

**Fig. 5:**
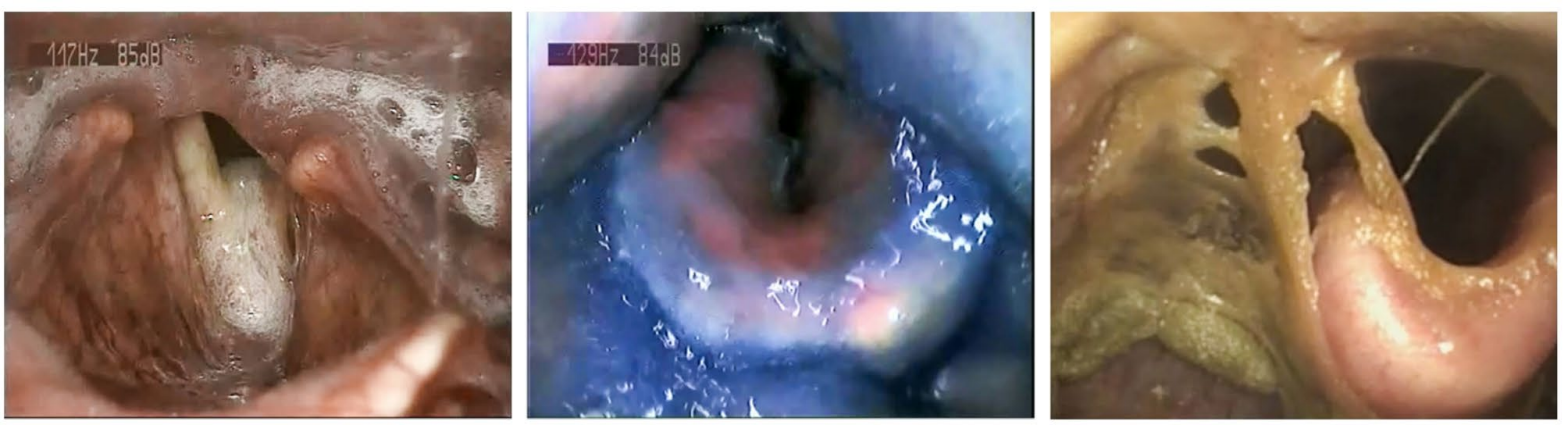
Laryngeal findings during flexible endoscopic evaluation of swallowing (FEES) in three PSD patients. Left: 65-year-old man with pseudohypersalivation and salivary aspiration. Center: 74-year-old woman with aspiration of Nutilis aqua (IDDSI level 4). Right: 79-year-old man on NPO showing compromised airway patency with dry, obstructive pharyngo-laryngeal secretions. IDDSI, International dysphagia diet standardisation initiative; PSD, post-stroke dysphagia.

When focusing on the 54 patients of the SAP group, 2 subjects developed pneumonia after day 14, thus making the acute stroke and dysphagia less relevant as primary influencing factors. However, in 5 (9.6%) of the remaining 52 patients, the chest X-ray was not clearly indicative of pneumonia, so the diagnosis of SAP was based on other symptoms. Treatment was palliative in 13.5% of SAP cases (n = 7). In addition to SLP diagnostics and swallowing therapy, a phoniatric assessment including flexible endoscopic evaluation of swallowing (FEES) was conducted in 9.6% of SAP patients (n = 5) to visualize the condition and function of the larynx with regard to oropharyngeal dysphagia. Figure 5 shows different endolaryngeal findings in three PSD patients on NPO.

**Fig. 6:**
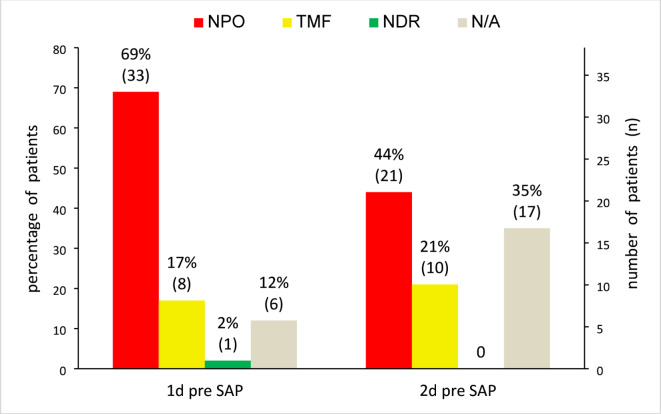
Feeding strategy 1 and 2 days before SAP diagnosis. d, day(s); N/A, not applicable, because of unknown feeding strategy; NDR, no dietary restrictions; NPO, nil per os; SAP, stroke-associated pneumonia; TMF, texture modified food.

Unfortunately, in 4 out of 52 cases, the exact date of the SAP diagnosis was not clearly documented. Among the remaining 48 patients, 81.3% developed SAP within the first three days, with an overall mean diagnosis day of 2.9 (SD = 2.9). Since SAP usually develops within the first 24 to 72 h after the stroke, we investigated the feeding status 2 days before SAP diagnosis date. On the day prior to the documented SAP occurrence, 68.8% of patients (33/48) had already been treated with NPO, 16.7% (8/48) were on a texture modified diet, 1 subject was on a normal diet, and 6 patients had an unclear dietary status (Fig. 6). The latter can be attributed to missing information, as these patients were not hospitalized on days 1 and/or 2 prior to pneumonia manifestation. Two days before SAP diagnosis, the type of diet could not be determined in 17 cases (35.4%), again due to early onset of pneumonia (between day 0 and 1). Additionally, 21 patients (43.8%) had NPO, 10 (20.8%) received TMF, and no patient was on a regular diet.

**Fig. 7:**
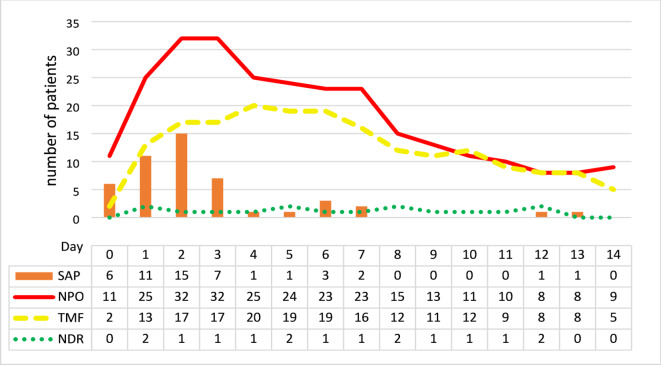
Daily feeding recommendation (NPO, TMF, NDR) and number of patients documented with SAP diagnosis during the first 2 weeks after hospitalization. NDR, no dietary restrictions; NPO, nil per os; SAP, stroke-associated pneumonia; TMF, texture modified food.

The majority of patients (31.3%) received the SAP diagnosis on day 2 (n = 15), despite having been on NPO status or TMF diet (Fig. 7). There were only a few cases within the SAP group that were on a regular diet (NDR). With regard to CSE- and FEES-based feeding recommendation, a day-by-day analysis throughout the first 2 weeks showed that the number of NPO patients increased up to the second day, remained stable on the third day, and then fell steadily. In contrast, the proportion of TMF patients revealed a delayed rise until day 4, before also falling. The increasing relative proportion of TMF compared to NPO patients over time indicates an improvement in general health and swallowing function.

### Secondary outcome parameters

SAP patients experienced a significantly higher incidence of nasogastric feeding tubes (*p* < 0.05) compared to those without SAP (Table 4). As expected, oral feeding on admission significantly reduced the likelihood of nasogastric tube placement (OR = 0.068, 95% CI 0.026–0.178, *p* < 0.001), based on logistic regression adjusted for the A^2^DS^2^ score. In contrast, a model including diet types (NPO, TMF, NDR) as predictors yielded no meaningful association (OR = 1.41 × 10^12^, *p* = 0.999).

**Table 4:** Secondary outcome parameters of the total PSD cohort, the non-SAP, and the SAP group.

	Total cohort (n = 160)	Non-SAP group (n = 106)	SAP group (n = 54)	*p*-value
LOS in days; mean (SD)	12.0 (8.6)	10.1 (6.5)	15.7 (10.8)	0.001
Nasogastric tubes	51 (31.9%)	28 (26.4%)	23 (42.6%)	0.038
Mortality	32 (20.0%)	17 (16.0%)	15 (27.8%)	0.079

LOS, length of hospital stay; PSD, post-stroke dysphagia; SAP, stroke-associated pneumonia.

Overall, 20% of the PSD cohort (32/160) died, with mortality numerically higher in the SAP group (27.8%, 15/54) compared to the non-SAP group (16.0%, 17/106); however, this difference did not reach statistical significance (*p* = 0.079). Of the deceased patients, 46.9% (15/32) had suffered pneumonia. Among the patients who passed away, 59.4% (n = 19) were on NPO, 37.5% (n = 12) received TMF, and only 1 patient (3.1%) had been recommended a non-restricted diet (NDR). The binary logistic regression for mortality with diet types as predictors, adjusted for the A^2^DS^2^ score as a confounder, revealed no significant associations (NPO: *p* = 0.507; TMF: *p* = 0.584; NDR: *p* = 0.790). In a multivariate model including individual confounders (i.e. NIHSS, atrial fibrillation, age, sex, and COPD), male sex (OR 3.559, 95% CI 1.409–8.991, *p* = 0.007) and age (OR 1.093, 95% CI 1.037–1.153, *p* = 0.001) emerged as significant risk factors for mortality. The mean age of the deceased subjects was 86 ± 8 years (mean ± SD) and thus significantly higher than that of survivors (78 ± 13, *p* = 0.003). In the SAP group, the average time of death occurred on day 17 of hospitalization (SD 18.1). Seven of these patients (46.7%) had already been given palliative care. Due to reduced vigilance in all seven cases, only 1 subject was able to partially receive TMF, while the others remained on NPO. NIHSS, pneumonia rate and diet on SLP admission did not differ significantly from the surviving patients.

SAP subjects had a significantly longer inpatient LOS (t-test, *p* < 0.001). The linear logistic regression for LOS with oral feeding and the A^2^DS^2^ score as predictors showed no significant associations (oral feeding: *p* = 0.496; A^2^DS^2^ score: *p* = 0.906). Likewise, an analysis including individual variables (age, sex, atrial fibrillation, NIHSS, COPD) did not identify any significant predictors, suggesting that other factors (e.g., availability of post-acute care) may account for differences in LOS.

## Discussion

### Interpretation of results

In our cohort, binary logistic regression revealed that NPO status was strongly associated with the development of SAP, with patients on NPO having approximately 2.5-fold higher odds of pneumonia compared to those on oral feeding. Stroke severity, measured by the NIHSS, was also a significant predictor, as were male sex and older age. Notably, the magnitude of the NPO effect in our dataset was comparable to, or greater than, several established clinical risk factors such as age and sex, highlighting the clinical relevance of feeding strategy in the early post-stroke period. The strong association between NPO and SAP in our dataset may reflect a higher baseline vulnerability of these patients: NPO is often prescribed for individuals with more severe strokes, reduced vigilance, or impaired airway protection—factors that themselves predispose to pneumonia. Stroke severity (NIHSS) showed an independent effect, consistent with the role of larger lesions, reduced mobility, and impaired protective reflexes in pneumonia pathogenesis. Male sex, also independently associated with SAP, has been linked in previous studies to higher aspiration risk and potentially to differences in immune response. The modest effect of age in our analysis is in line with the general observation that advanced age is a marker for frailty and reduced pulmonary reserve, which can compound the impact of other risk factors.

Taken together, these findings underline a fundamental paradox in dysphagia management: patients placed on NPO to prevent aspiration were in our analysis among those with the highest risk of developing SAP. This phenomenon has also been noted in prior studies and likely reflects the fact that NPO is used in patients already at highest baseline risk—those with severe neurological impairment, reduced consciousness, compromised airway protection, and poor oral hygiene. Many PSD patients in our cohort were diagnosed with pneumonia before starting an oral diet, with most diagnoses occurring by day three, consistent with previous international research^5,36^. This supports the growing recognition of the “NPO paradox”, which has increasingly been cited by experts in the field as a reflection of underlying patient vulnerability rather than the presence or absence of oral feeding itself^5,28,37–39^. However, although our findings suggest an association between dietary restrictions and pneumonia risk, the potential for confounding must be considered and is further addressed in the Limitations section below.

### SAP pathophysiology

Given that acute stroke patients frequently develop pneumonia despite being kept NPO, it is crucial to investigate the various risk factors that contribute to the development of deep airway disease beyond just aspiration of food^40,41^. Our study confirms the predictive power of the A^2^DS^2^ score and its associated risk factors, as previously established in the literature^15,16,42^. The results highlight that pathophysiological mechanisms following an acute stroke, such as systemic and pulmonary inflammation, as well as stroke-induced immunosuppression, substantially increase the risk of SAP. A multicenter study^14^ revealed that stroke-induced immunosuppression plays a critical role in the development of stroke-associated pneumonia (SAP), alongside aspiration. That study reported a 5.2% incidence of pneumonia among 486 acute stroke patients and established a link between SAP and immunosuppression due to sympathetic hyperactivity following a stroke, which impairs immune defenses. Notably, dysphagia significantly increased SAP risk only in patients with low immune competence, as indicated by mHLA-DR levels^43,44^. Post-stroke neuroinflammation initiates systemic immunosuppression, leading to a reduction in lymphocytes and the deactivation of monocytes, thereby compromising antibacterial defenses^45–47^.

### Predictive factors and clinical tools

Other risk factors for SAP include stroke severity, dysphagia, impaired consciousness, mechanical ventilation, advanced age, and male gender^27,48,49^. The known gender-specific differences in stroke are confirmed in our cohort: men were on average 8 years younger than women, and more frequently found in the SAP group. In addition, preventive antibiotics have not proven effective in reducing SAP incidence, further emphasizing the role of immunosuppression in post-stroke infections^13,14,50–53^. Poor oral hygiene combined with the progressive loss of swallowing ability further enhances the risk of pneumonia in these patients^54,55^. Maintaining good oral hygiene has been associated with a reduced risk of pneumonia, decreased dependence on tube feeding, and improved overall oral health in stroke patients^56^.

### Clinical implications and feeding strategies

Besides oral hygiene, great importance is attributed to oral feeding^57^. In our study, TMF was not statistically associated with a reduced incidence of SAP. Regardless of this lack of impact on pneumonia risk, TMF plays an important role in maintaining oral intake and enhancing swallowing function. It provides patients with dysphagia the opportunity to consume oral food despite impaired swallowing function^58–60^. In our study, TMF enabled 57% of non-SAP und 43% of SAP patients to eat or receive oral diet until discharge (or the day of death) despite dysphagia. The IDDSI standard facilitates the objective classification of food textures and enhances communication within the multidisciplinary team and further treating professions^30,61–63^. This aligns with the paradigm shift in dysphagia therapy, moving away from a conservative approach that strictly avoids aspiration toward greater patient self-determination and the incorporation of neuroplastic treatment strategies^64–66^.

The principles of “use it or lose it” and “use it and improve it” are particularly applicable to swallowing functions. Neuronal connections that are not utilized may deteriorate, while active training can enhance relevant neural pathways and improve swallowing abilities, especially post-stroke^67–69^. The goal is to maintain and enhance residual swallowing capacity by providing adapted foods and drinks or at least water, which poses minimal pulmonary toxicity. To promote self-determination, patients and their families should be informed about the benefits and risks associated with various nutritional options, allowing them to make informed decisions^70,71^.

Risk-feeding strategies, i.e. oral intake despite the risk of aspiration, are currently under discussion^72^. Risk-feeding may be appropriate in situations where quality of life is prioritized, alternative nutrition offers limited benefit, and an informed, interdisciplinary decision supports the patient’s values and clinical context. One example of such a strategy is the ice chip protocol, which enables patients to consume small pieces of ice orally, showing promise as a safe and effective rehabilitation tool for dysphagia^73^. The Frazier Free Water Protocol^74–76^ is another water-based intervention that has demonstrated positive effects on hydration, compliance, and quality of life. However, caution is warranted in cases with severely impaired consciousness, poor airway protection, or FEES-confirmed silent aspiration, where the risk of significant pulmonary complications may outweigh the potential benefits. Moreover, the Frazier Free Water Protocol has only been evaluated in a small, narrowly defined patient population, underscoring the need for further studies with broader inclusion criteria.

### Study limitations

However, our bicenter study findings must be interpreted in light of several limitations. Due to its retrospective design that extends over a longer period of time, various uncontrolled factors may have influenced the cohorts. Furthermore, the diagnosis of SAP can be particular challenging due to non-specific symptoms, difficulties in obtaining sputum samples, overlapping causes of hypoxia, unreliable infection markers, inconclusive chest X-rays, inconsistent diagnostic criteria, and the limited reliability of biomarkers like procalcitonin^15,35,50,52,77^. The incidence of dysphagia also varies considerably depending on the screening methods utilized^6^. Although the CSE was performed by experienced SLPs in adherence to established guidelines, its validity is limited, and the number of more objective assessments (e.g. FEES) was relatively low. This can be partially attributed to the high proportion of patients who were not ready for oral nutrition due to insufficient vigilance (37.7%). These findings are consistent with recent data from Reitz et al. (2024), who reported that 50% of SLP-assessed patients in the intensive care unit were prescribed NPO because of impaired consciousness^34^. Finally, it also remains unclear whether the patients who have a high pneumonia rate under NPO would not also suffer from this if they had received TMF or NDR. Given that the observed association between NPO status and SAP may be confounded by stroke severity and related clinical factors, our findings should be interpreted as exploratory. Larger studies with comprehensive, multivariable-adjusted analyses are needed to validate these results and better account for potential confounders. Future research should also incorporate objective dysphagia diagnostics (e.g., FEES or videofluoroscopy) and stratify patients by the severity of swallowing disorder or aspiration risk to enhance data quality and allow for more precise analysis of diet-related effects.

## Conclusion

If oral abstinence from food can be minimized by implementing appropriate dysphagia diets, this might positively impact both the SAP rate and the rehabilitation of dysphagia. Patients on NPO status, in particular, have an increased SAP risk, necessitating effective preventive measures for this population. As such, it is crucial to communicate the importance of oral hygiene to all professionals and caregivers involved in patient care. For individuals who fulfill the requirements for oral nutrition, i.e. are sufficiently vigilant, it should be assessed whether the negative consequences of NPO can be avoided by basal oral intake on a water basis. Oral abstinence from food is often perceived as a significant burden by many patients and should be carefully considered as an option. In general, upcoming investigations should focus on prospective trials comparing standardized TMF protocols and time-limited NPO with early individualized swallowing interventions to clarify their impact on SAP and overall clinical outcomes. In this context, randomized controlled trials investigating the effects of a cautious water-based diet on pneumonia risk and recovery from dysphagia in aspiration-prone PSD patients are strongly recommended and are already being planned by the authors of this study.

## Data Availability

The datasets compiled and investigated for this study are not publicly available, as they contain, for example, information that could jeopardize the privacy of research participants, but can be obtained from the corresponding author on reasonable request.
